# Supplementary material for: Low Vitamin C Status in Patients with Cancer Is Associated with Patient and Tumor Characteristics
Source: Nutrients. 2020 Aug 5;12(8):2338. doi: 10.3390/nu12082338 (PMC7468872; doi:10.3390/nu12082338)
Supplement: Supplementary file 1 [file nutrients-12-02338-s001.pdf]

Table S1: Characteristics of the two cohorts of patients with cancer.

	Pre-surgical cohort		Therapy cohort [a]	
	**total**	**fasting**	**total**	**fasting**
**Participants (n=)**	99	81	100	69
**Sex (% females)**	61.6	61.0	62.0	56.5
**Ethnicity (% Maori/Pacific)**	9.1	11.0	6.0	8.7
**Age (years, mean ±SEM)**	64.0 ± 1.2	63.6 ± 1.4	59.6 ± 1.4	58.7 ± 1.7
**BMI (mean ±SEM)**	29.9 ± 0.7	30.4 ± 0.8	28.6 ± 0.7	28.6 ± 0.9
**Smoking (% current smokers)**	5.1	4.9	11.0	13.0
**Exercise (% doing <60min/week)**	23.2	24.4	26.0	29.0
**Ascorbate intake (mg/day, mean ±SEM)**	61.5 ± 5.7	61.0 ± 6.2	82.8 ± 14.4	61.9 ± 6.7
**(mg/day, median)**	42.0	43.0	53.9	46.9
**Supplement use (% taking supplements)** [b]	28.3	18.3	32.0	18.8
**High dose ascorbate infusion (%)** [c]	1.0	0	4.0	1.4
**Plasma ascorbate (μM, mean ±SEM)**	65.4 ± 3.1	57.2 ± 2.7	55.1 ± 3.0	46.8 ± 3.2
**(μM, median)**	60.8	58.4	53.6	44.6
**Cancer type (%)**				
**Breast**	35.4	32.9	36.0	31.9
**Colorectal**	20.2	19.5	18.0	17.4
**Prostate**	10.1	12.2	3.0	4.3
**Skin**	2.0	2.4	17.0	14.5
**Endometrial**	7.1	8.5		
**Urothelial**	6.1	7.3	1.0	1.4
**Lymphoma**			6.0	7.2
**Other**	19.2	17.2	19.0	23.3
**Tumour stage (TNM)**				
**Stage 1/2**	58	48	28	19
**Stage 3**	24	19	18	11
**Stage 4/Metastasis/Recurrence**	13	12	47	39
**missing**	4	2	7	0

[a]Therapy cohort includes patients treated with chemotherapy and immunotherapy; [b]Supplement use is self-reported and includes 'not regularly', 'fairly regularly' and 'today'; [c]High dose ascorbate infusion includes all those who have ever had an infusion for any reason and at any dose.
